# The Phosphodiesterase Type 5 Inhibitor Sildenafil Improves DNA Stability and Redox Homeostasis in Systemic Sclerosis Fibroblasts Exposed to Reactive Oxygen Species

**DOI:** 10.3390/antiox9090786

**Published:** 2020-08-25

**Authors:** Luigi Di Luigi, Guglielmo Duranti, Ambra Antonioni, Paolo Sgrò, Roberta Ceci, Clara Crescioli, Stefania Sabatini, Andrea Lenzi, Daniela Caporossi, Francesco Del Galdo, Ivan Dimauro, Cristina Antinozzi

**Affiliations:** 1 Unit of Endocrinology, Department of Movement, Human and Health Sciences, University of Rome “Foro Italico”, Piazza Lauro de Bosis 15, 00135 Rome, Italy; luigi.diluigi@uniroma4.it (L.D.L.); paolo.sgro@uniroma4.it (P.S.); clara.crescioli@uniroma4.it (C.C.); 2 Unit of Biochemistry, Department of Movement, Human and Health Sciences, University of Rome “Foro Italico”, Piazza Lauro de Bosis 15, 00135 Rome, Italy; guglielmo.duranti@uniroma4.it (G.D.); roberta.ceci@uniroma4.it (R.C.); stefania.sabatini@uniroma4.it (S.S.); 3 Unit of Biology and Genetics, Department of Movement, Human and Health Sciences, University of Rome “Foro Italico”, Piazza Lauro de Bosis 15, 00135 Rome, Italy; ambra.anto@hotmail.it (A.A.); daniela.caporossi@uniroma4.it (D.C.); ivan.dimauro@uniroma4.it (I.D.); 4 Department of Experimental Medicine, Sapienza University of Rome, Viale Regina Elena 324, 00161 Rome, Italy; andrea.lenzi@uniroma1.it; 5 Division of Rheumatic and Musculoskeletal Diseases, Leeds Institute of Molecular Medicine, University of Leeds, Woodhouse, Leeds LS2 9JT, UK; F.DelGaldo@leeds.ac.uk

**Keywords:** oxidative stress, sildenafil, DNA damage, systemic sclerosis

## Abstract

Systemic sclerosis (SSc) is a multi-system connective tissue disease characterized by the increased deposition of extracellular matrix proteins such as collagen and fibronectin. Although the pathogenesis is not completely understood, a number of studies suggest that free radicals could be the major contributors to the disease. Indeed, different studies demonstrated how oxidative stress could contribute to the fibrotic process activation at the level of the skin and visceral organs. Emerging evidences highlight the beneficial effects of sildenafil, a phosphodiesterase type 5 inhibitor (PDE5i), which protects different cell lines from the cell damage induced by reactive oxygen species (ROS). These data make sildenafil a good candidate for therapeutic treatment aimed to protect biological macromolecules against oxidative damage, thus preserving cell viability. The purpose of this study was to evaluate the sensitivity of SSc dermal fibroblasts to an oxidative insult and the ability for sildenafil to prevent/reduce the DNA damage due to ROS action. Additionally, we evaluated the capacity for sildenafil to influence redox homeostasis and cytotoxicity, as well as cell proliferation and cell cycle progression. We demonstrated that SSc fibroblasts have an increased sensitivity to a pro-oxidant environment in comparison to healthy controls. The sildenafil treatment reduced ROS-induced DNA damage, counteracted the negative effects of ROS on cell viability and proliferation, and promoted the activity of specific enzymes involved in redox homeostasis maintenance. To our knowledge, in this report, we demonstrate, for the first time, that sildenafil administration prevents ROS-induced instability in human dermal fibroblasts isolated by SSc patients. These results expand the use of PDE5i as therapeutic agents in SSc by indicating a protective role in tissue damage induced by oxidative insult.

## 1. Introduction

Systemic sclerosis (SSc) is a multi-system connective tissue disorder characterized by the increased deposition of extracellular matrix proteins, where increased oxidative stress plays a role in the activation of the fibrotic process at the level of the skin and visceral organs [1,2,3].

In fact, it is known that excessive oxidative stress contributes to vascular damage, jeopardizes the function of the endothelial system, leading to immune system involvement, and it participates in the establishment and maintenance of fibroblast activation [4,5].

Several authors have shown that SSc patients have a reduced antioxidant capacity; however, the exact stage of the disease at which the increase in reactive oxygen species (ROS) occurs is still uncertain [6,7]. The plasma levels of ascorbic acid, α-tocopherol, β-carotene, and selenium were found lower in SSc patients than in healthy controls [6,7]. Moreover, patients with SSc have shown a high rate of chromosomal breakages, consistent with the known clastogenic activity of ROS, and increased levels of plasma markers of oxidative stress [8,9]. In particular, several authors have demonstrated that the systemic oxidative imbalance occurring in SSc patients induces changes in different subcellular components and macromolecules, oxidative denaturation, and derangement, as well as the loss of lipid asymmetry in membranes and DNA damage [10,11]. These are considered cellular changes that can induce premature cell senescence or cell death.

Controversial results exist about the possible beneficial effects of the treatment with different antioxidants in patients with SSc. The favorable outcome depends on the nature of the antioxidant substance, stage of the disease, and the duration of treatment [12,13,14,15,16,17,18].

Emerging evidences suggest specific protective effects of phosphodiesterase type 5 (PDE5) inhibition with sildenafil in protecting them from genotoxic damage induced by ROS in different cell lines [19,20,21]. Phosphodiesterases (PDE) are a group of ubiquitous enzymes that hydrolyze the nucleotides cyclic adenosine monophosphate (cAMP) or cyclic guanosine monophosphate (cGMP) to their inactive forms AMP and GMP [22]. In mammalians, PDE enzymes are classified into 11 families—namely, PDE1-PDE11. The use of PDE5 inhibitors (PDE5i) has been established in Raynaud’s phenomenon and pulmonary arterial hypertension, and they are emerging as antifibrotic agents in interstitial lung disease [23,24,25].

The high potential antioxidant capability of sildenafil [26,27] makes this PDE5 inhibitor a good candidate for therapeutic treatment aimed to reduce oxidative damage to biological macromolecules, thus preserving cell viability.

The objective of this study was to evaluate the sensitivity of SSc dermal fibroblasts to oxidative stress and the ability for sildenafil to prevent/reduce the DNA damage induced by ROS. Additionally, we evaluated the capacity of sildenafil to influence redox homeostasis and cytotoxicity, as well as cell proliferation and cell cycle progression. To our knowledge, this is the first study on the redox characterization of SSc fibroblasts and their response to ROS-induced DNA damage, where it also highlights the possible capacity of sildenafil to determine an increased protection against oxidative stress-related alterations

## 2. Materials and Methods

### 2.1. Chemicals

Dulbecco’s modified Eagle’s medium (DMEM)/Ham’s F-12 medium (1:1) (with and without phenol red), phosphate-buffered saline (PBS) Ca^2+^/Mg^2+^-free, bovine serum albumin (BSA) fraction V, glutamine, antibiotics, collagenase type IV, NaOH, Bradford reagent, 4′,6-Diamidino-2-phenylindole (DAPI), hydrogen peroxide, sildenafil citrate salts (98%), propidium iodide, ribonuclease enzyme, a redox-sensitive probe, and all reagents for Western blotting were from Sigma Aldrich (St. Louis, MO, USA). Fetal calf serum was from Gibco^®^ (Grand Island, NY, USA). 2-mercaptoethanol was from Life Technologies, Inc. Laboratories (Grand Island, NY, USA). Cy3-labeled secondary antibodies (Abs) were from Jackson Laboratory (Bar Harbor, ME, USA); peroxidase secondary Abs and all reagents for Sodium Dodecyl Sulphate-PolyAcrylamide Gel Electrophoresis (SDS-PAGE) were from Millipore (Billerica, MA, USA). Plastic ware for cell cultures and disposable filtration units for growth media preparation were purchased from Corning (Milan, Italy). 

### 2.2. Cell Culture

Human dermal fibroblasts (Hfbs) were isolated at the SSc clinic within the Leeds Institute of Rheumatic and Musculoskeletal Medicine (Leeds, UK) and processed as previously described [28]. Cells were derived from excisional skin biopsies of 3 patients with early diffuse cutaneous SSc (SSc) (mean age 61.9 ± 9.2 years) and 3 healthy controls (H) (mean age 55.6 ± 8.0 years) [28]. Informed consent was obtained and approved by the National Research Ethics Service (NRES) Committee (REC 10/H1306/88). The combined treatment with sildenafil and hydrogen peroxide was performed by adding this PDE5i to the culture medium 0.5 h before treatment with the pro-oxidant. The sildenafil concentration (1 µM) was chosen on the basis of the near-therapeutic doses utilized to treat erectile dysfunction, according to its pharmacokinetics (maximum drug concentration, Cmax and area under the time–concentration curves, AUC) [29].

### 2.3. Protein Expression Analysis

Healthy and SSc fibroblasts were treated for 24 h with H_2_O_2_ (50 and 100 µM) in the presence or absence of sildenafil. Cells were then lysed in Radioimmunoprecipitation assay (RIPA) buffer (150 mM NaCl, 50 mM tris-HCl pH 8, 1 mM Ethylenediaminetetraacetic acid (EDTA), 1% nonyl phenoxypolyethoxylethanol (NP40), 0.25% sodium deoxycholate, and 0.1% SDS, water to volume) supplemented with protease and phosphatase inhibitor cocktails (Sigma-Aldrich, Darmstadt, Germany).

For the immunoblot analysis, an equal amount of proteins (20–30 μg) was resolved in SDS-polyacrylamide (Bio-Rad Laboratories, Inc., Hercules, CA, USA) gels and transferred on polyvinylidene fluoride (PVDF) or nitrocellulose membranes, as previously described [30,31]. Thereafter, membranes were incubated with primary antibodies (Abs) appropriately diluted in Tween Tris-buffered saline (TTBS), followed by peroxidase-conjugated secondary immunoglobulin (Ig)G (1:10,000). Proteins were revealed by the enhanced chemiluminescence system (ECL plus, Millipore, Burlington, MA, USA). Image acquisition was performed with Image Quant Las 4000 software (GE Healthcare, Chicago, IL, USA). Densitometric analysis was performed with Quantity One^®^ software version 4.6.8 (Bio-Rad Laboratories, Inc., Hercules, CA, USA). Western blot analysis was performed for three/four independent experiments. The list of primary antibodies utilized for the Western blot is reported in Table 1.

### 2.4. Immunofluorescence Microscopy

A total of 1 × 10^4^ cells were seeded onto glass coverslips in the growth medium. For γH2aX and RAD51 localization, healthy and SSc fibroblasts were treated with sildenafil and then stimulated for 0.5 h or 1 h with 50 µM or 100 µM H_2_O_2_. After stimulation, cells were washed out from the drugs and maintained in a drug-free medium for 4 h. Next, cells were washed and fixed with 95% ethanol and 5% acetic acid for 5 min and incubated with a blocking buffer containing 3% BSA/TBS for 30 min at room temperature. Primary Abs were incubated 1 h in blocking buffer, followed by the Cy3-conjugated secondary Ab (1.500). For method specificity, slides lacking the primary Abs were processed. Details on the primary antibodies are reported in Table 1. DAPI nucleic acid stain (1:10,000) was used to mark nuclei. Images were acquired at the magnification of 60×, and slides were examined with a Zeiss Z1 microscope (Zeiss International, Oberkochen, Germany) and Leica TCS SP2 (Leica, Milano, Italy). Experiments were performed three times.

### 2.5. 3-(4,5-Dimethylthiazol-2-yl)-2,5-Diphenyl-2H-Tetrazolium Bromide (MTT) Assay

Healthy and SSc fibroblasts (4 × 10^3^ cells/well) were seeded in 96-well plates and incubated 1 h (T0) or 24 h (T24) with 50 µM or 100 µM H_2_O_2_ in the presence or absence of sildenafil. As previously described [32], the MTT assay was performed using Cell Proliferation Kit I (MTT) (Roche, Basilea, Switzerland), according to the manufacturer’s instructions. Experiments were performed in triplicate with different cell preparations.

### 2.6. Cell Viability

For the cell viability assay, healthy and SSc fibroblasts were seeded in 6-well plates and incubated for 1 h with 50 µM or 100 µM H_2_O_2_ in the presence or absence of sildenafil. At the end of the incubation, drugs were washed out, and cells were either harvested (T0) or cultured for 7 (7 d) and 14 days (14 d) in drug-free media. Cell viability was assessed each day by trypan blue (0.05% *v/v* solution in PBS) exclusion mixed in a ratio of 1:1. Cell number counting was assayed by a hemocytometer, and only cells excluding trypan blue dye were analyzed. The number of viable cells at each time point was derived, averaging at least five different fields for each well. Each experimental point was repeated in duplicate or triplicate. Results are expressed as the number of cells. Experiments were performed four times with different cell preparations.

### 2.7. Cell Cycle Analysis

To evaluate the cell cycle progression, 25 × 10^4^ healthy and SSc fibroblasts were seeded, with or without sildenafil, and then stimulated for 24 h with 50 µM or 100 µM H_2_O_2_. Propidium iodide (PI) assay was performed as previously described [33]. Briefly, cells were harvested, washed with PBS, suspended into 50% fetal bovine serum (FBS)-PBS solution, and fixed with 70% (*v/v*) alcohol at −20 °C overnight. After centrifugation, cells were suspended with 50 µM PI solution and ribonuclease A. DNA content was analyzed with FACScan CytoFLEX (Beckman Coulter, Brea, CA, USA). Experiments were performed in triplicate with different cell preparations.

### 2.8. Measurement of Intracellular ROS Levels

For intracellular ROS levels analysis, healthy and SSc fibroblasts were seeded at 5 × 10^3^/mL in 6-well plates and incubated for 1 h with H_2_O_2_ 50 µM or 100 µM in the presence or absence of sildenafil. At the end of treatment, cells were loaded with 200 µL of 5 µM dichloro-dihydro-fluorescein diacetate (DCFH-DA) dissolved in dimethylsulfoxide (DMSO) in PBS solution and maintained at 37 °C in the dark. The loading buffer was removed after 40 min, and the cells were incubated at 37 °C for an additional 30 min in a prewarmed growth medium. Then, the cell layer was washed with serum-free DMEM, and the level of ROS was determined by a Spark^®^ fluorescence plate reader (Biocompare, South San Francisco, CA, USA) at 488/525 nm. Results obtained were expressed as absorbance arbitrary units/mg of proteins tested.

### 2.9. Thiobarbituric Acid Reactive Substances (TBARS)

TBARS levels were assayed by spectrophotometric analysis, as previously described [34]. The methodology calculates malondialdehyde (MDA) and other aldehydes generated by lipid peroxidation induced by hydroxyl free radicals. Briefly, healthy and SSc fibroblasts, treated with sildenafil and then stimulated for 1 h with 50 µM or 100 µM H_2_O_2_, were lysed in RIPA buffer. Cell lysate (150 μL) was added to 25 μL 0.2% butylated hydroxytoluene (BHT) and 600 μL of 15% aqueous trichloroacetic acid (TCA). Then, the mixture was centrifuged at 4000× *g* for 15 min at 4 °C. The deproteinized supernatant was transferred in a cryotube and added with 600 μL of thiobarbituric acid (TBA, 0.375% in 0.25 M HCl). Samples were then heated at 100 °C for 15 min in boiling water. The absorbance was determined at 535 nm by a spectrophotometric method and compared to standard MDA (1,1,3,3-tetramethoxypropane) solution. The levels of TBARS were expressed as nmol/mg of proteins.

### 2.10. Glutathione Homeostasis

Intracellular reduced (GSH) and oxidized (GSSG) glutathione amounts were evaluated by a 5,5’-dithio-bis(2-nitrobenzoic acid) (DTNB)-glutathione reductase recycling assay, as previously described [35]. Briefly, 1 × 10^7^ healthy and SSc fibroblasts, treated with sildenafil and then stimulated for 1 h with 50 µM or 100 µM H_2_O_2_, were collected and suspended in 1:1 (*v/v*) 5% sulfosalicylic acid (SSA). Cells were lysed by freezing and thawing and, then, were centrifuged at 10,000× *g* for 5 min at 4 °C. The deproteinized supernatant was analyzed for the total glutathione content. Oxidized glutathione (GSSG) was measured in samples where reduced GSH was masked by pretreatment with 2-vinylpyridine (2%). Ten microliters of the sample were added to the reaction buffer (700 μL nicotinamide adenine dinucleotide phosphate (NADPH) (0.3 mM), 100 μL DTNB (6 mM), and 190 μL H_2_O). The reaction was started by adding 2.66 U/mL glutathione reductase and followed at 412 nm by the TNB stoichiometric formation. Samples Δ optical density (ΔOD)/min412 were compared to those obtained by using glutathione standards. Results were normalized for protein contents.

### 2.11. Enzymatic Activities

Healthy and SSc fibroblasts were treated with sildenafil and then stimulated for 1 h with 50 µM or 100 µM H_2_O_2_. After treatment, cells were immediately collected. Intracellular superoxide dismutase (SOD) and catalase (CAT) activities were measured using commercial assay kits (Cayman Chemical Company, Ann Arbor, MI, USA), as previously described [35]. Experiments were performed at different times using different cell preparations. Results obtained as units/mg of proteins tested were expressed as fold over controls.

### 2.12. Cytokine Assay

Healthy and SSc fibroblasts were plated at 2 × 10^4^ cells/mL in 96-well tissue culture plates and incubated 1 h with 50 µM or 100 µM H_2_O_2_ in the presence or absence of sildenafil. Cell culture supernatants were assayed for the macrophage migration inhibitor factor (MIF) by the magnetic bead-based multiplex assay according to the manufacturer’s protocol. As previously described [36], data acquisition was performed by the Bio-Plex 200 System™ (Bio-Rad Laboratories, Inc., Hercules, CA, USA). Data analysis was performed by Bio-Plex Manager™ 6.0 software (Bio-Rad Laboratories, Inc., Hercules, CA, USA). Quality control pools of low, normal, and high concentrations for all parameters were included in each assay. Data were expressed as pg/mL. Cells supernatants were run in triplicate.

### 2.13. Statistical Analysis

The statistical analysis was conducted using GraphPad Prism software 8.0 (GraphPad Software, San Diego, CA, USA). To test the normality of the quantitative variables, the Kolmogorov-Smirnov or Shapiro-Wilk tests were applied. Normally distributed, continuous variables were analyzed using one- or two-way ANOVA and a Student’s *t*-test. In all cases, *p*-value ≤ 0.05 was considered significant. All data were expressed as the mean ± SE.

## 3. Results

### 3.1. SSc Fibroblasts Appeared More Sensitive to Oxidative Stress-Induced DNA Double-Strand Breaks (DSBs) in Comparison to the Healthy Control Cells

**Figure 1:**
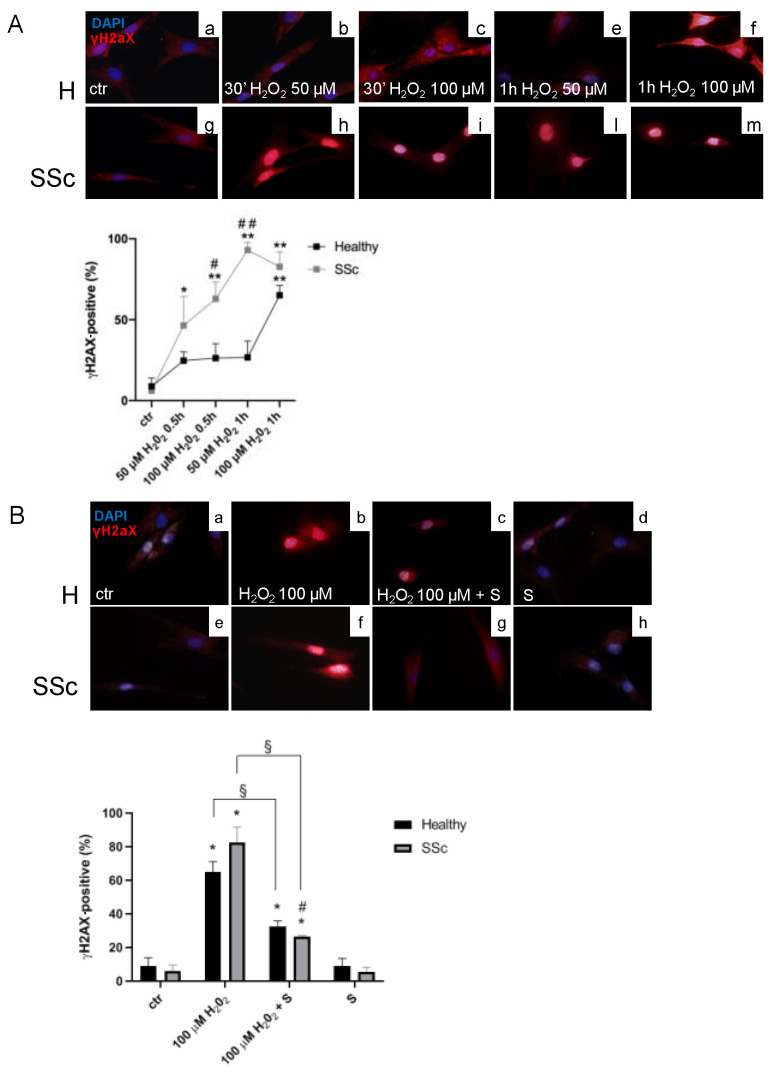
Analysis of H2aX phosphorylation in healthy controls (H) and systemic sclerosis (SSc) fibroblasts exposed to a pro-oxidant environment. (**A**) Immunofluorescence analysis of γH2aX in H and SSc fibroblasts treated with 50 and 100 µM H_2_O_2_ for 0.5 and 1 h. (**B**) Exposure of H and SSc fibroblasts to the highest dose of H_2_O_2_ (100 µM for 1 h) in the presence or in absence of sildenafil (1 µM). Nuclei were stained with 4′,6-diamidino-2-phenylindole (DAPI) (blue). Pictures are representative of at least three separate experiments; magnification 60×. Diagrams represent the percentage of γH2aX-positive cells in the healthy controls (black lines/columns) and SSc (grey lines/columns) fibroblasts. * *p* < 0.05, ** *p* < 0.01 vs. ctr, ^#^ *p* < 0.05, ^##^ *p* < 0.01 healthy vs. SSc, and ^§^ *p* < 0.05 H_2_O_2_ vs. H_2_O_2_ + S. Data represent mean ± SE. Results are derived from three separate experiments using different cell preparations. S, sildenafil.

The immunofluorescence analysis demonstrated that, already to the lowest doses tested (50 µM H_2_O_2_ for 0.5 h), SSc Hfbs showed higher sensitivity to oxidative stress compared to healthy ones, with a percentage of γH2aX-positive cells of 46.4 ± 17.9% after 30 min (*p* < 0.05 vs. control (ctr)), reaching 93.0 ± 5.7% after 1 h (*p* < 0.01 vs. ctr and *p* < 0.01 vs. healthy) (Figure 1A). At the highest dose of the pro-oxidant (100 µM H_2_O_2_), SSc Hfbs showed increased γH2aX by 62.9 ± 12.7% after 30 min (*p* < 0.01 vs. ctr and *p* < 0.05 vs. healthy) and 82.7 ± 9.1% after 1 h (*p* < 0.01 vs. ctr) (Figure 1A). Differently, healthy Hfbs showed a significant increase of γH2aX by 65.1 ± 6.1% only after 1 h with the H_2_O_2_ 100 µM (*p* < 0.01 vs. ctr) (Figure 1A).

### 3.2. Sildenafil Improved the DNA Repair Activation Process in H_2_O_2_-Damaged Healthy and SSc Fibroblasts

**Figure 2:**
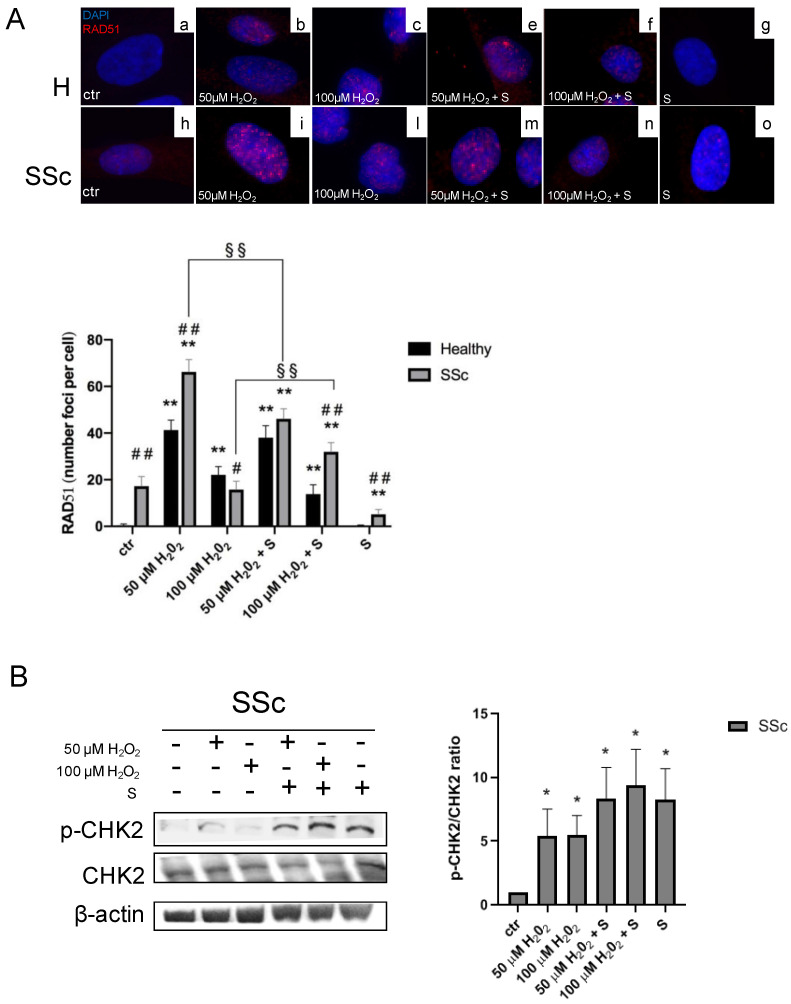
Analysis of DNA repair ability in healthy control (H) and SSc fibroblasts exposed to a pro-oxidant environment. (**A**) Representative immunofluorescence analysis of RAD51 in H and SSc fibroblasts treated with 50 and 100 µM H_2_O_2_ for 1h in the presence or in absence of sildenafil 1 µM. The diagram represents the number of RAD51 foci per cell in healthy (black columns) and SSc (grey columns) fibroblasts. (**B**) Western blot analysis for a serine/threonine kinase (CHK2) phosphorylation in SSc fibroblasts. Diagram depicted a densitometric analysis expressed as the ratio p-CHK2/total CHK2. * *p* < 0.05, ** *p* < 0.01 vs. ctr, ^#^ *p* < 0.05, ^##^ *p* < 0.01 healthy vs. SSc, ^§§^ *p* < 0.01 H_2_O_2_ vs. H_2_O_2_ + S. Data represent mean ± SE. Results are derived from three separate experiments using different cell preparations. S, sildenafil.

To assess the effects of sildenafil on γH2AX induced by the higher dose of hydrogen peroxide, we performed an immunofluorescence analysis in healthy and SSc Hfbs treated with H_2_O_2_ 100 µM for 1 h in the presence or not of sildenafil. As shown in Figure 1B, a pro-oxidant environment induced a significant increase of γH2AX in both healthy (ctr vs. 100 µM H_2_O_2_: 8.8% ± 5.2% vs. 65.5 ± 12.2%, *p* < 0.05) and SSc fibroblasts (ctr vs. 100 µM H_2_O_2_: 6.2 ± 3.6% vs. 82.7 ± 9.1%, *p* < 0.05); however, the presence of sildenafil significantly decreased the percentage of γH2AX-positive cells by 32.8 ± 4.9% in healthy (100 µM H_2_O_2_ vs. 100 µM H_2_O_2_ + S, 65.5 ± 12.2% vs. 32.7 ± 3.5%, *p* < 0.05) and 56.1 ± 0.9% in SSc fibroblasts (100 µM H_2_O_2_ vs. 100 µM H_2_O_2_ + S, 82.7 ± 9.1% vs. 26.6 ± 0.7%, *p* < 0.05), respectively. When the same analysis was extended to RAD51, a protein involved in DNA repair mechanisms [37], we found that, at the basal condition, SSc already showed a higher number of RAD51 foci per cell than healthy Hfbs (healthy vs. SSc: 0.5 ± 0.2 vs. 17.2 ± 4.2, *p* < 0.01) (Figure 2A). Exposure to 50 µM of H_2_O_2_ induced a significant increase of RAD51 foci per cell either in healthy (ctr vs. 50 µM H_2_O_2_: 0.5 ± 0.2 vs. 41.3 ± 4.2, *p* < 0.01) or in SSc fibroblasts (ctr vs. 50 µM H_2_O_2_: 17.2 ± 4.2 vs. 66.3 ± 5.3, *p* < 0.01), although the number of RAD51 foci was significantly higher in the SSc (*p* < 0.01). Of interest, the highest dose of H_2_O_2_ (100 µM) induced major recruitment of RAD51 only in healthy Hfbs (ctr vs. 100 µM H_2_O_2_: 0.5 ± 0.2 vs. 22.1 ± 3.6 foci/cell, *p* < 0.01) (Figure 2A). The pretreatment with sildenafil reduced significantly the recruitment of RAD51 in the presence of low doses of H_2_O_2_ (50 µM) only in SSc Hfbs (50 µM H_2_O_2_ vs. 50 µM H_2_O_2_ + S: 66.3 ± 5.3 vs. 46.1 ± 4.3, *p* < 0.01), whereas no differences were observed in healthy Hfbs, where the level of RAD51 remained quite similar to that without sildenafil (*p* > 0.05) (Figure 2A). At high doses of H_2_O_2_ (100 µM), the presence of sildenafil increased RAD51 recruitment exclusively in SSc Hfbs (100 µM H_2_O_2_ vs. 100 µM H_2_O_2_ + S: 15.8 ± 3.6 vs. 32.0 ± 3.9, *p* < 0.01). No significant changes were observed in healthy Hfbs exposed to the pro-oxidant condition between the presence and absence of sildenafil (*p* > 0.05). Finally, the analysis of serine/threonine kinase (CHK2) modulation, a key component of the DNA damage response, highlighted that, in a pro-oxidant environment (50 µM and 100 µM H_2_O_2_ for 24 h), there was an increase of its phosphorylated form in SSc Hfbs (50 µM H_2_O_2_: 5.4 ± 2.1 and 100 µM H_2_O_2_: 5.5 ± 1.5, *p* < 0.05), independently of the presence of sildenafil (*p* > 0.05) (Figure 2B). No significant effects were demonstrated in healthy Hfbs (data not shown).

### 3.3. Sildenafil Sustained the Cell Viability and Ameliorated the Cell Proliferation in SSc Fibroblasts

**Figure 3:**
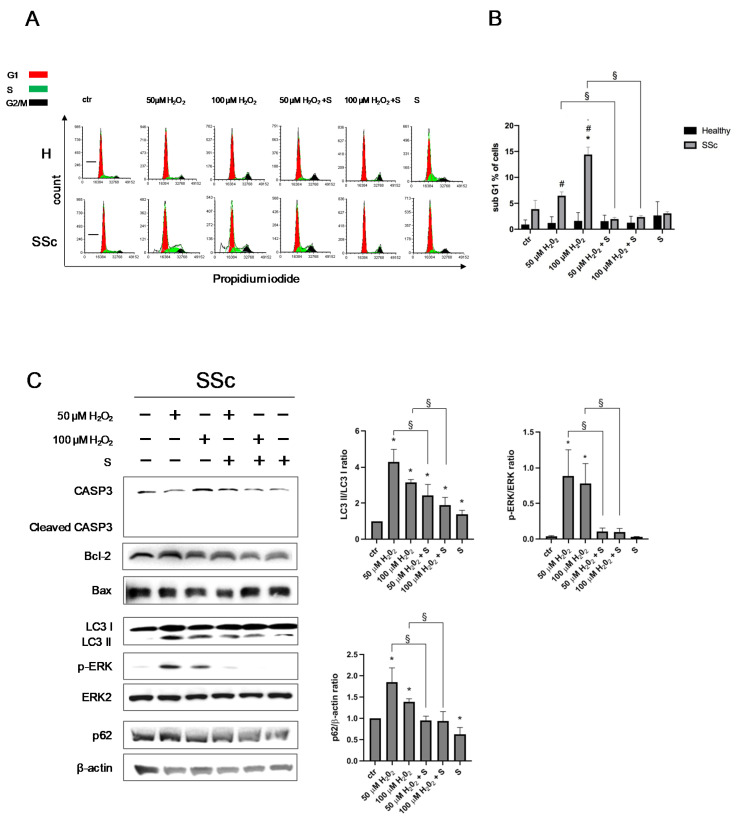
Analysis of the cell viability in healthy control (H) and SSc fibroblasts exposed to a pro-oxidant environment. (**A**) The cell cycle progression of H and SSc fibroblasts stimulated with 50 and 100 µM H_2_O_2_ for 24 h in the presence or in absence of sildenafil 1 µM. (**B**) Histogram represented the percentage of cells in the G1 subphase in healthy (black columns) and in SSc (grey columns) fibroblasts. (**C**) At the same experimental conditions, CASP3, cleaved CASP3, Bcl-2, Bax, and LC3 I and II, as well as ERK2, p-ERK, and P62, were analyzed in SSc fibroblasts though immunoblotting. Histograms depicted the densitometric analysis (mean ± SE) expressed as ratios of LC3 II/LC3 I, p-ERK/total ERK2, and P62/β-actin. * *p* < 0.05 vs. ctr, ^#^ *p* < 0.05 healthy vs. SSc, and ^§^ *p* < 0.05 H_2_O_2_ vs. H_2_O_2_ + S. S, sildenafil, and CASP3, CASPASE3.

To evaluate the effect of sildenafil on the cell cycle progression following H_2_O_2_ administration (50 µM and 100 µM H_2_O_2_ for 24 h), a flow cytometric analysis was performed. As shown in Figure 3A and Figure S1, at each experimental point, no significant differences in the various phases of the cell cycle were observed in both types of Hfbs (*p* > 0.05). However, in SSc, the highest dose of pro-oxidant induced an increase of cells blocked in the G1 sub-phase (6.5% after H_2_O_2_ 50 µM, *p* > 0.05 and 14.4% after H_2_O_2_ 100 µM, *p* < 0.05). Furthermore, the pretreatment with sildenafil reduced the proportion of cells in the G1 subphase by 77% after H_2_O_2_ 50 µM (from 6.5% to 1.9%, *p* < 0.05) and by 83% after H_2_O_2_ 100 µM (from 14.4% to 2.4%, *p* < 0.05) (Figure 3B).

To better characterize this phenomenon, specific protein markers of the apoptotic and autophagic processes were analyzed. As shown in Figure 3C, at the same experimental conditions, there was an increase of the cleaved form of LC3B (LC3 II), p62 expression, and ERK phosphorylation levels (*p* < 0.05). No significant effects were observed for CASPASE 3 cleavage, Bcl-2, or Bax (*p* > 0.05). The presence of sildenafil reduced the activation of LC3 II, the level of p62, and ERK phosphorylation (*p* < 0.05).

**Figure 4:**
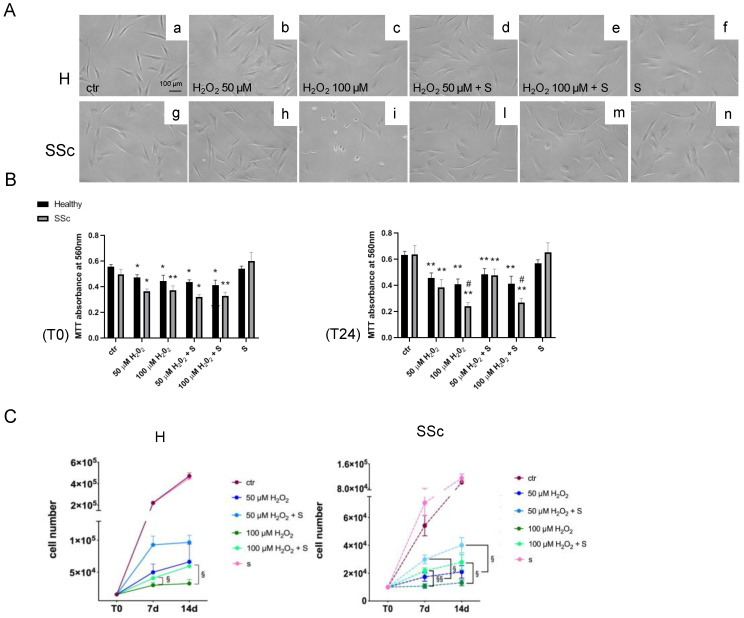
Analysis of the cell proliferation in healthy control (H) and SSc fibroblasts exposed to a pro-oxidant environment. (**A**) Morphological changes in H and SSc fibroblasts at the basal level (panels a,g) treated with 50 (panels b,h) and 100 µM H_2_O_2_ (panels c,i) for 24 h in the presence or in absence of sildenafil 1 µM (panels d–f,l–n). Scale Bar = 100 µm. (**B**) Cell viability was measured in healthy (black columns) and SSc fibroblasts (grey columns) stimulated with 50 and 100 µM H_2_O_2_ for 1 h (T0, left panel) and 24 h (T24, right panel) in the presence or in absence of sildenafil 1 µM. (**C**) Cell proliferation analysis in H (left panel) and SSc fibroblasts (right panel) treated with 50 and 100 µM H_2_O_2_ for 1 h in the presence or in absence of sildenafil 1 µM at the basal level (T0) after 7 days (7 d) and 14 days (14 d) of washing from the treatment. * *p* < 0.05, ** *p* < 0.01 vs. ctr, ^#^ *p* < 0.05 healthy vs. SSc, ^§^ *p* < 0.05, and ^§§^ *p* < 0.01 H_2_O_2_ vs. H_2_O_2_ + S. Data are expressed as mean ± SE. S, sildenafil.

A further analysis of cell proliferation at T0 and T24, on both types of fibroblasts, highlighted a generalized reduction of cell proliferation following H_2_O_2_ exposure (*p* < 0.05 and *p* < 0.01) independently from the presence of sildenafil, although at 24 h, the presence of sildenafil showed a trend towards improving the cell proliferation (*p* = 0.0523) (Figure 4A,B). Long-term analysis of the cell viability after 7 d and 14 d demonstrated a different effect between the experimental groups (Figure 4C). In healthy Hfbs, the most significant effect of sildenafil was observed with the higher (100 µM) H_2_O_2_ exposure at 7 d (H_2_O_2_ vs. H_2_O_2_ + S: (2.9 ± 0.5) × 10^4^ vs. (4.1 ± 0.7) × 10^4^, *p* < 0.05) and 14 d (H_2_O_2_ vs. H_2_O_2_ + S: (3.2 ± 0.7) × 10^4^ vs. (5.9 ± 0.1) × 10^4^, *p* < 0.05), whereas, in SSc Hfbs, it was observed with both H_2_O_2_ doses at 7 d (50 µM H_2_O_2_ vs. 50 µM H_2_O_2_ + S: (1.7 ± 0.5) × 10^4^ vs. (3.0 ± 0.4) × 10^4^, *p* < 0.05 and 100 µM H_2_O_2_ vs. 100 µM H_2_O_2_ + S: (1.0 ± 0.2) × 10^4^ vs. (2.2 ± 0.3) × 10^4^, *p* < 0.01) and 14 d (50 µM H_2_O_2_ vs. 50 µM H_2_O_2_ + S: (2.3 ± 0.6) × 10^4^ vs. (4.0 ± 0.6) × 10^4^, *p* < 0.05 and 100 µM H_2_O_2_ vs. 100 µM H_2_O_2_ + S: (1.3 ± 0.3) × 10^4^ vs. (2.8 ± 0.8) × 10^4^, *p* < 0.05) (Figure 4C).

### 3.4. Sildenafil Reduces the ROS Levels in Healthy and SSc Fibroblasts

Previous experiments have been performed to demonstrate that sildenafil per se did not retain scavenger activity on oxygen-derived free radicals when added to the culture medium (data not shown).

**Figure 5:**
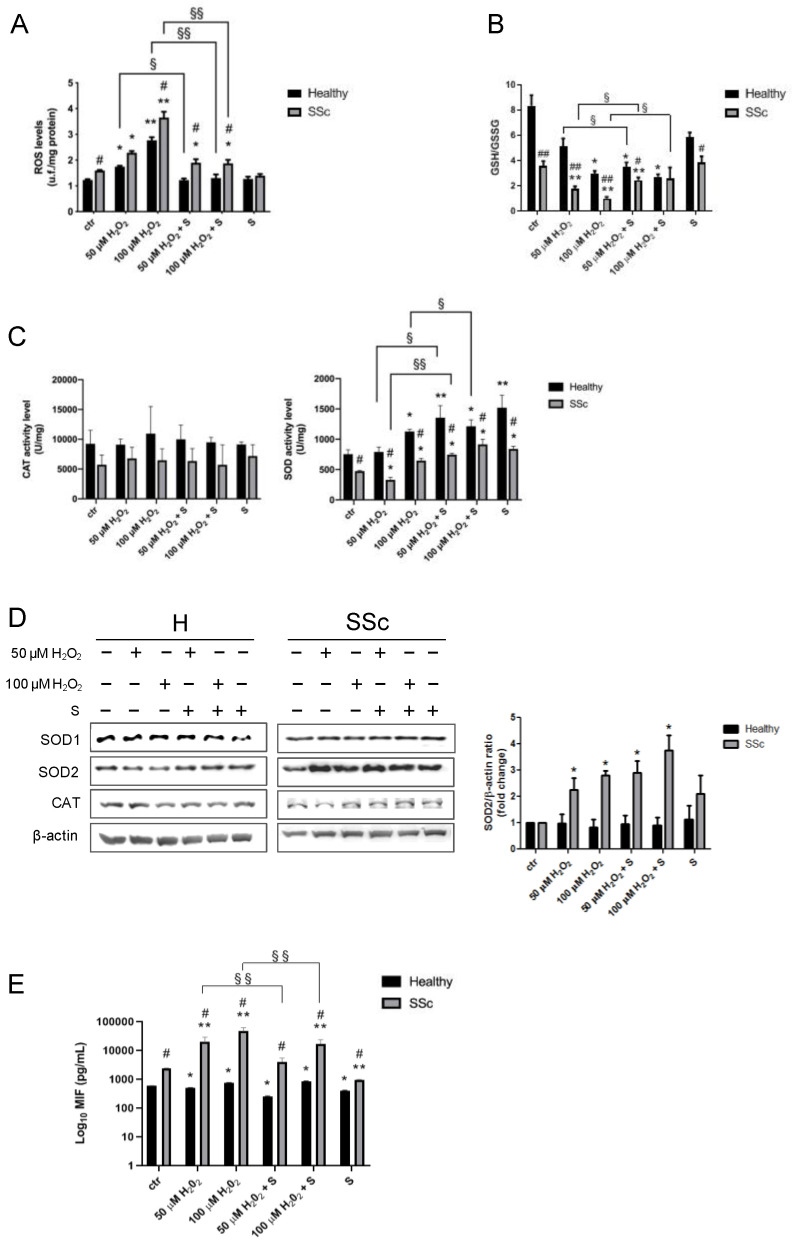
Analysis of the redox status in healthy control (H) and SSc fibroblasts after hydrogen peroxide administration. Healthy (black columns) and SSc (grey columns) fibroblasts were treated for 1 h with 50 and 100 µM H_2_O_2_ in the presence or in absence of sildenafil 1 µM and then analyzed for (**A**) ROS amount; (**B**) GSH/GSSG; (**C**) catalase (CAT) and superoxide dismutase (SOD) activity; (**D**) CAT, SOD1, and SOD2 protein contents; and (**E**) MIF level. * *p* < 0.05, ** *p* < 0.01 vs. ctr, ^##^ *p* < 0.01, ^#^ *p* < 0.05 healthy vs. SSc, ^§^ *p* < 0.05, and ^§§^ *p* < 0.05 H_2_O_2_ vs. H_2_O_2_ + S. Data are expressed as mean ± SE. S, sildenafil; ROS, reactive oxygen species; GSSG, glutathione oxidized states; GSH, glutathione reduced states; SOD1 and 2, manganese superoxide dismutase isoforms 1 and 2; and MIF, macrophage migration inhibitory factor.

As shown in Figure 5A, already at the basal level, SSc showed higher intracellular ROS levels when compared to healthy fibroblasts (healthy vs. SSc: 1.2 ± 0.0 vs. 1.6 ± 0.0, *p* < 0.05). Hydrogen peroxide administration induced a significant dose-dependent increase in ROS levels, particularly in SSc fibroblasts (healthy, 50 µM H_2_O_2_: 1.7 ± 0.0, *p* < 0.05 vs. ctr and 100 µM H_2_O_2_: 2.8 ± 0.1, *p* < 0.01 vs. ctr and SSc, 50 µM H_2_O_2_: 2.3 ± 0.1, *p* < 0.05 vs. ctr and 100 µM H_2_O_2_: 3.7 ± 0.2, *p* < 0.01 vs. ctr). Interestingly, the combined treatment with sildenafil reduced H_2_O_2_-induced ROS levels in healthy fibroblasts at both doses tested (50 µM H_2_O_2_ vs. 50 µM H_2_O_2_ + S: 1.7 ± 0.0 vs. 1.2 ± 0.1, *p* > 0.05 and 100 µM H_2_O_2_ vs. 100 µM H_2_O_2_ + S: 2.8 ± 0.1 vs. 1.3 ± 0.1, *p* < 0.01), whereas, in SSc cells, there was a significant reduction of ROS only at the highest dose (100 µM H_2_O_2_ vs. 100 µM H_2_O_2_ + S: 3.7 ± 0.2 vs. 1.9 ± 0.1, *p* < 0.01), respectively. No significant differences were found with sildenafil administration alone (*p* > 0.05) (Figure 5A).

### 3.5. Sildenafil Ameliorates an Antioxidant Response in SSc Fibroblasts

The evaluation of the glutathione homeostasis revealed significant differences in the GSH/GSSG ratio between the experimental groups. In particular, in SSc, the total glutathione levels were already found lower compared with the counterpart healthy ones at the basal level (healthy vs. SSc: 164.9 ± 8.6 vs. 109.3 ± 5.8, *p* < 0.01) (Figure S2A), also showing a greater reduction of the GSH/GSSG ratio when exposed to both concentrations of H_2_O_2_ (ctr vs. 50 µM H_2_O_2_: 3.6 ± 0.3 vs. 1.7 ± 0.2, *p* < 0.01 and ctr vs. 100 µM H_2_O_2_: 3.6 ± 0.3 vs. 0.9 ± 0.1, *p* < 0.01). Differently, healthy Hfbs showed a significant reduction of the GSH/GSSG ratio only after exposure to the highest dose of H_2_O_2_ (ctr vs. 50 µM H_2_O_2_: 8.3 ± 0.8 vs. 5.1 ± 0.6, *p* > 0.05 and ctr vs. 100 µM H_2_O_2_: 8.3 ± 0.8 vs. 2.9 ± 0.3, *p* < 0.05) (Figure 5B). Pretreatment with sildenafil has proved particularly effective in SSc fibroblasts where it counteracts the reduction of the GSH/GSSH ratio induced by free radicals (50 µM H_2_O_2_ vs. 50 µM H_2_O_2_ + S: 1.7 ± 0.2 vs. 2.4 ± 0.2, *p* < 0.05 and 100 µM H_2_O_2_ vs. 100 µM H_2_O_2_ + S: 0.9 ± 0.1 vs. 2.6 ± 0.8, *p* < 0.05) (Figure 5B). Both types of fibroblasts showed no difference after the administration of sildenafil alone (*p* > 0.05). At the level of the antioxidant enzyme activities, we found substantial differences between two types of fibroblasts depending on the enzyme examined (Figure 5C). In particular, while catalase (CAT) activity showed only a tendency to be lower in SSc cells compared to the healthy counterparts (*p* = 0.0938), and its level was not influenced by the presence of H_2_O_2_ and/or sildenafil (*p* > 0.05), the analysis of superoxide dismutase (SOD) activity highlighted distinct and significant differences between the two groups. Indeed, SOD activity was ≈ 37.7% lower in SSc compared with the counterpart healthy (ctr: healthy vs. SSc, 754.9 ± 41.3 vs. 473.8 ± 6.2, *p* < 0.05). In the presence of ROS, both cell lines were responsive, with a significant increase of SOD activity only at the higher dose (healthy, 100 µM H_2_O_2_: 1127.8 ± 21.3 and SSc, 100 µM H_2_O_2_ 647.2 ± 19.9, *p* < 0.05). Moreover, while in healthy ones, the combined treatment with sildenafil induced a major increase in SOD activity in both H_2_O_2_ doses tested (50 µM H_2_O_2_ vs. 50 µM H_2_O_2_ + S: 791.0 ± 46.7 vs. 1356.4 ± 112.9, *p* < 0.05 and 100 µM H_2_O_2_ vs. 100 µM H_2_O_2_ + S: 1127.8 ± 21.3 vs. 1213.8 ± 61.5, *p* < 0.05), in SSc, this increase was present only at the low dose (50 µM H_2_O_2_ vs. 50 µM H_2_O_2_ + S: 330.0 ± 22.5 vs. 745.7 ± 12.3, *p* < 0.05) (Figure 5C). The presence of sildenafil alone induced a significant increase in SOD activity in both cell types compared with unstimulated cells (healthy, ctr vs. S: 754.9 ± 41.3 vs. 1521.2 ± 11.7, *p* < 0.01 and SSc, ctr vs. S: 473.8 ± 6.2 vs. 839.8 ± 24.9, *p* < 0.05).

At the protein level, no effects were detected for CAT and SOD1 (*p* > 0.05), whereas SOD2 was significantly upregulated only in SSc cells exposed to H_2_O_2_ (*p* < 0.05), independently from the presence of sildenafil (*p* > 0.05) (Figure 5D).

### 3.6. Macrophage Migration Inhibitory Factor Level

As already demonstrated by Kim et al. (2008) [38], the analysis of the macrophage migration inhibitory factor (MIF) revealed already at the basal level profound differences, with SSc cells showing higher levels compared with the healthy control (healthy vs. SSc: 586.3 ± 5.1 vs. 2448.0 ± 30.1, *p* < 0.05) (Figure 5E). The presence of hydrogen peroxide evocated in our cellular models a different response of this inflammatory cytokine. In particular, we found a slight decrease of MIF in healthy ones (50 µM H_2_O_2_: 508.8 ± 3.8, *p* < 0.05 and 100 µM H_2_O_2_: 749.5 ± 24.8, *p* < 0.05) and a massive increase in SSc ones (50 µM H_2_O_2_: 19,685.0 ± 9135.0, *p* < 0.01 and 100 µM H_2_O_2_: 47,417.0 ± 15,026.0, *p* < 0.01) (Figure 5E). Interestingly, the concomitant treatment with sildenafil produced significant effects only in SSc cells reducing the MIF secretion induced by hydrogen peroxide (50 µM H_2_O_2_ vs. 50 µM H_2_O_2_ + S: 19,685.0 ± 9135.0 vs. 4127.0 ± 1367.0, *p* < 0.01 and 100 µM H_2_O_2_ vs. 100 µM H_2_O_2_ + S: 47,417.0 ± 15,026.0 vs. 17,285.0 ± 6374.0, *p* < 0.01) (Figure 5E).

### 3.7. Sildenafil Mitigates the Effect of Oxidative Insult on TBAR Levels

As shown in Figure S2C, no differences in the TBAR levels were observed at the basal level between healthy and SSc cells (*p* > 0.05). Under pro-oxidant conditions, TBAR levels increased in both cell types tested, showing the greatest sensitivity of SSc. Indeed, already at a low dose of H_2_O_2_, there was a significant increase in TBAR in SSc cells (ctr vs. 50 µM H_2_O_2_: 0.6 ± 0.0 vs. 0.8 ± 0.0, *p* < 0.01). Only at the highest concentration of hydrogen peroxide, there was a significant increase in TBAR in both cell types (healthy, ctr vs. 100 µM H_2_O_2_: 0.5 ± 0.0 vs. 0.9 ± 0.1, *p* < 0.01 and SSc, ctr vs. 100 µM H_2_O_2_: 0.6 ± 0.0 vs. 1.0 ± 0.1, *p* < 0.01). The concomitant presence of sildenafil induced no significant variation in TBAR levels after the low dose of H_2_O_2_ (*p* > 0.05), whereas, at the highest dose, sildenafil reported TBARS levels similar to the control in both experimental groups (*p* < 0.01). No significant differences were found with sildenafil administration alone (*p* > 0.05).

## 4. Discussion

The present study demonstrates for the first time that the PDE5 inhibitor sildenafil reduces the sensitivity to DNA damage of human SSc fibroblasts exposed to a pro-oxidant environment, improving their genomic stability. In addition, sildenafil counteracts the negative effects of ROS on cell viability and proliferation, inhibiting the activation of the autophagic pathway and modulating the activity of specific enzymes involved in redox homeostasis. Taken together, these results suggest that sildenafil may be a possible candidate for therapeutic treatment aimed at counteracting oxidative stress adverse effects.

There is considerable evidence implicating sildenafil as a key molecule capable of preventing ROS-induced DNA damage in several different types of cells, a condition present during the progression of many diseases—as, for example, in atherosclerosis, cardiovascular diseases, and systemic sclerosis [7,20,21,39].

In the present study, we utilized a cell culture of human dermal fibroblasts from healthy and SSc patients already validated as a useful model to investigate the mechanisms involved in SSc fibrosis [23,40,41]. We found that SSc fibroblasts were more sensitive to exogenous exposure to ROS compared with the healthy counterparts. In fact, a brief exposure to low concentrations of hydrogen peroxide (50 µM) already induced an increased nuclear accumulation of γH2aX, a marker of single and double DNA stranded breaks (SSBs and DSBs) [42]. Moreover, we observed that the accumulation of γH2aX induced by the high dose of hydrogen peroxide (100 µM) both in healthy and in SSc ones was less evident in the presence of sildenafil. Therefore, as already suggested by others in different pathological conditions [19,20,21], here, we showed that sildenafil reduced ROS-induced DNA damage in SSc and healthy fibroblasts. Additionally, our data demonstrated that the presence of sildenafil modifies the nuclear distribution of RAD51, a central player in homologous recombination (HR) and DSBs repair [43,44]. The RAD51 redistribution to chromatin and nuclear foci formations, mainly induced by DSBs, is crucial to HR repair. In particular, the formation of RAD51 nuclear foci was dramatically enhanced by H_2_O_2_, indicating the proceeding of HR repair; however, and more markedly in SSc cells, sildenafil modified ROS-induced effects. In particular, in the presence of low levels of DNA damage, the PDE5i determined a reduced redistribution of RAD51. Presumably, the extent of DNA damage did not exceed the intrinsic capacity of the cells to repair themselves. Differently, in the presence of massive DNA damage, sildenafil improved the cellular response by increasing the efficiency of cell repair systems.

CHK2 is a checkpoint kinase and a key component of the DNA damage response regulating the binding of RAD51 to DNA [45,46]. Genotoxic stress triggering CHK2 phosphorylation regulates a variety of downstream effectors, inducing a proper cellular response as cell cycle checkpoint activation [47], cell death, and DNA repair [48,49]. The analysis of the cell cycle progression, along with the analysis of specific markers of cell death, further highlighted the greater sensitivity of SSc fibroblasts to ROS insult. In particular, our results suggested that these cells were unstable, less proliferative, and with a significant number of cells that undergo autophagic cell death compared to the healthy cells. Among the beneficial pleiotropic effects of sildenafil, its ability to preserve the cell cycle kinetics under oxidative stress conditions has already been demonstrated in vivo and in vitro on different cell lines [20,21,50,51,52]; however, this is the first study showing that sildenafil treatment rescued SSc cells from the sub-G1 phase, restoring their normal cell cycle distribution and protecting them from autophagy. The lack of effect of sildenafil on CHK2 modulation was not surprising, since it could already be maximally activated by other cellular pathways responsive to ROS [53]. All these data were further confirmed by the data on cell proliferation, showing a partial recovery when sildenafil was concomitant to oxidative stress (Figure 4).

There are evidences of antioxidative effects of sildenafil on different cell lines and animal models [19,20,21,26,27,54,55,56], and very recently, our group has shown that sildenafil was involved in ROS-mediated signaling through the modulation of Signal transducer and activator of transcription (STAT) 3, ERK, nuclear factor kappa-light-chain-enhancer of activated B cells (NF-Κb), and protein kinase B (PKB/AKT), as well as in reducing the expression/secretion of proinflammatory and profibrotic cytokines in SSc fibroblasts exposed to H_2_O_2_ [23]. Here, for the first time, we successfully demonstrated that the presence of sildenafil ameliorates the management of redox imbalance in SSc fibroblasts exposed to a pro-oxidant environment, reducing ROS levels and improving the efficiency of the glutathione system, as well as increasing the level of SOD and its activity. These results were further supported by the reduced production of both the macrophage inhibitory factor (MIF), a pleiotropic inflammatory cytokine with broad target cell specificity that is secreted following several stimuli, including oxidative stress [57], and thiobarbituric acid reactive substances (TBARS), formed as a bioproduct of lipid peroxidation [58].

The molecular mechanism behind the pharmacologic actions of this PDE5i relates to its potential to increase intracellular cyclic guanosine monophosphate (cGMP) [59]. The regulations of cell function by extrinsic factors occurs through a series of second-messenger signals starting by a ligand-receptor interaction and, then, modulating both the intensity and the nature of immediate and delayed cellular responses. Thus, second messengers exert control over principal cellular events. There is evidence that biological responses triggered by oxidative products are able to provoke various pathogenic intracellular signals involving calcium, G-proteins, cAMP, cGMP, phospholipase C and D, protein kinase C, ceramide, and the mitogen-activated protein kinase (MAP) kinase cascade, leading to cellular dysfunction [60]. Thus, as suggested by Abdollahi et al. (2003) [56], increasing cyclic nucleotides by the use of phosphodiesterase inhibitors could overcome oxidative stress-induced cellular dysfunctions. To confirm this hypothesis, recently, it has been demonstrated that the augmentation of the cGMP/Protein Kinase G (cGMP/PKG) pathway ameliorated the antioxidant response of retinal cells cultured in hypoxic conditions, increasing the superoxide dismutase and catalase enzyme activities [61]. However, considering the complexity of cellular oxidative stress associated with this pathology, it stands to reason that a complete mechanism of action for sildenafil could require additional cellular pathways involved in the regulation of inflammation, proliferation, cell death, and antioxidant defenses [62,63,64].

## 5. Conclusions

Although our in vitro system represents a simplified model reproducing the alteration of the redox homeostasis determined by the pathological state [65,66,67], we believe that our research extends the scope of sildenafil as a therapeutic agent in oxidative stress-related pathologies for SSc.

**Figure 6:**
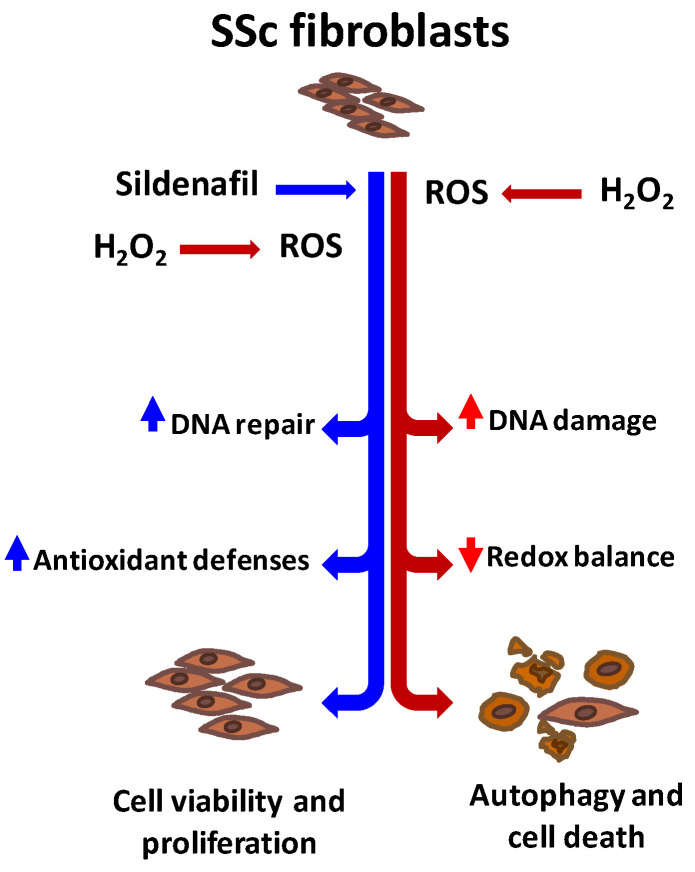
Reactive oxygen species (ROS) resulting from pro-oxidant exposure (H_2_O_2_, red arrows) increase the DNA damage and affect the redox homeostasis in systemic sclerosis fibroblasts (SSc). As a consequence, cells undergo cell death by autophagy. In the same pro-oxidant environment, the presence of the phosphodiesterase type 5 inhibitor sildenafil (blue arrows) counteracts the negative effects of ROS on cell viability and proliferation, inhibiting the activation of the autophagic pathway and modulating the activity of specific enzymes involved in redox homeostasis.

Particularly, our findings offer compelling evidences indicating that the administration of sildenafil in SSc fibroblasts exposed to a pro-oxidant: (i) protects against ROS-induced DNA damage, (ii) preserves the cell cycle kinetics, and (iii) improves redox homeostasis (Figure 6).

Therefore, the aforementioned effects suggest a possible positive impact of sildenafil not only on the treatment of the first signs of the disease, such as endovascular damage and decreased blood flow in the digital vein (Raynaud’s phenomenon), but, also, on the last stages when skin fibrosis occurs.

However, further studies are needed to deepen the molecular mechanisms by which sildenafil acts as an antigenotoxic drug and antioxidant, as well as to establish the feasibility and efficacy of this PDE5i in the clinical setting of patients at risk of developing SSc and in those where the pathology is already in progress.

## Figures and Tables

**Table 1:** List of primary antibodies utilized.

Antigen	Product Number	Dilution	Manufacturer
Superoxide dismutase (SOD) 1	sc-101523	1:1000	Santa Cruz (Santa Cruz, CA, USA)
Superoxide dismutase (SOD) 2	sod-110	1:1000	StressGen (San Diego, CA, USA)
Protein light chain (LC3) I-II	sc-271625	1:1000	Santa Cruz
Phospho-checkpoint kinase (p-CHK) 2	2197	1:1000	Cell Signaling (Leiden, The Netherlands
Checkpoint kinase (CHK) 2	sc-5278	1:1000	Santa Cruz
Caspase (CASP) 3	sc-56053	1:1000	Santa Cruz
BCL2 associated X (BAX)	sc-526	1:1000	Santa Cruz
B-cell lymphoma-2 (BCL-2)	50E3	1:1000	Cell signaling
Phospho-extracellular signal-regulated kinases (p-ERK) 1/2	sc-7383	1:1000	Santa Cruz
Extracellular signal-regulated kinases (ERK) 2	sc-154	1:1000	Santa Cruz
Sequestosome 1 (P62)	sc-48402	1:1000	Santa Cruz
Catalase (CAT)	sc-271803	1:1000	Santa Cruz
β-actin	sc-47778	1:5000	Santa Cruz
γ-histone family member X (γH2AX)	ab26350	1:100	Abcam (Cambridge, UK)
DNA repair protein RAD51 (RAD51)	ab63801	1:1000	Abcam

## Supplementary Materials

The following are available online at https://www.mdpi.com/2076-3921/9/9/786/s1: Figure S1: Analysis of the cell cycle progression of healthy control (H) and SSc fibroblasts exposed to a pro-oxidant agent. Figure S2: Analysis of the redox status in healthy control and SSc fibroblasts treated with hydrogen peroxide.
