# Supplementary material for: The Phosphodiesterase Type 5 Inhibitor Sildenafil Improves DNA Stability and Redox Homeostasis in Systemic Sclerosis Fibroblasts Exposed to Reactive Oxygen Species
Source: Antioxidants (Basel). 2020 Aug 25;9(9):786. doi: 10.3390/antiox9090786 (PMC7555932; doi:10.3390/antiox9090786)
Supplement: Supplementary file 1 [file antioxidants-09-00786-s001.pdf]

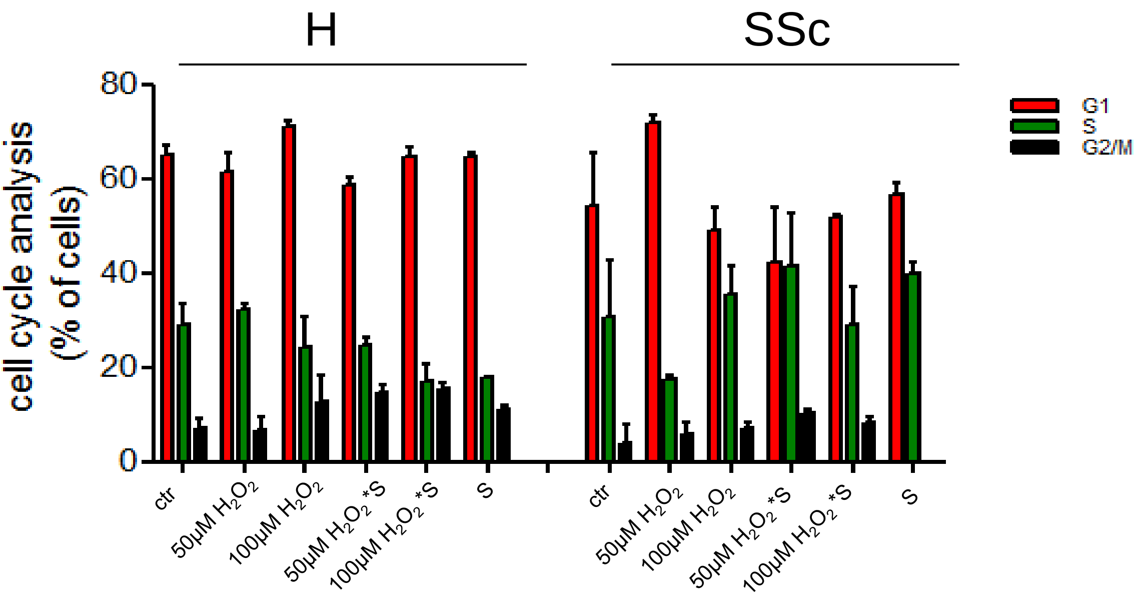

Figure S1. Analysis of cell cycle progression of healthy control (H) and SSc fibroblasts exposed to a pro-oxidant agent. Healthy and SSc fibroblasts were treated for 24h with 50 and 100μM H_2O_2 in presence or in absence of sildenafil 1μM and then analyzed for cell cycle distribution in G1 (red columns), S (green columns), and G2/M phases (black columns). Data are expressed as percentage (%) ±SE of cells. S, sildenafil.

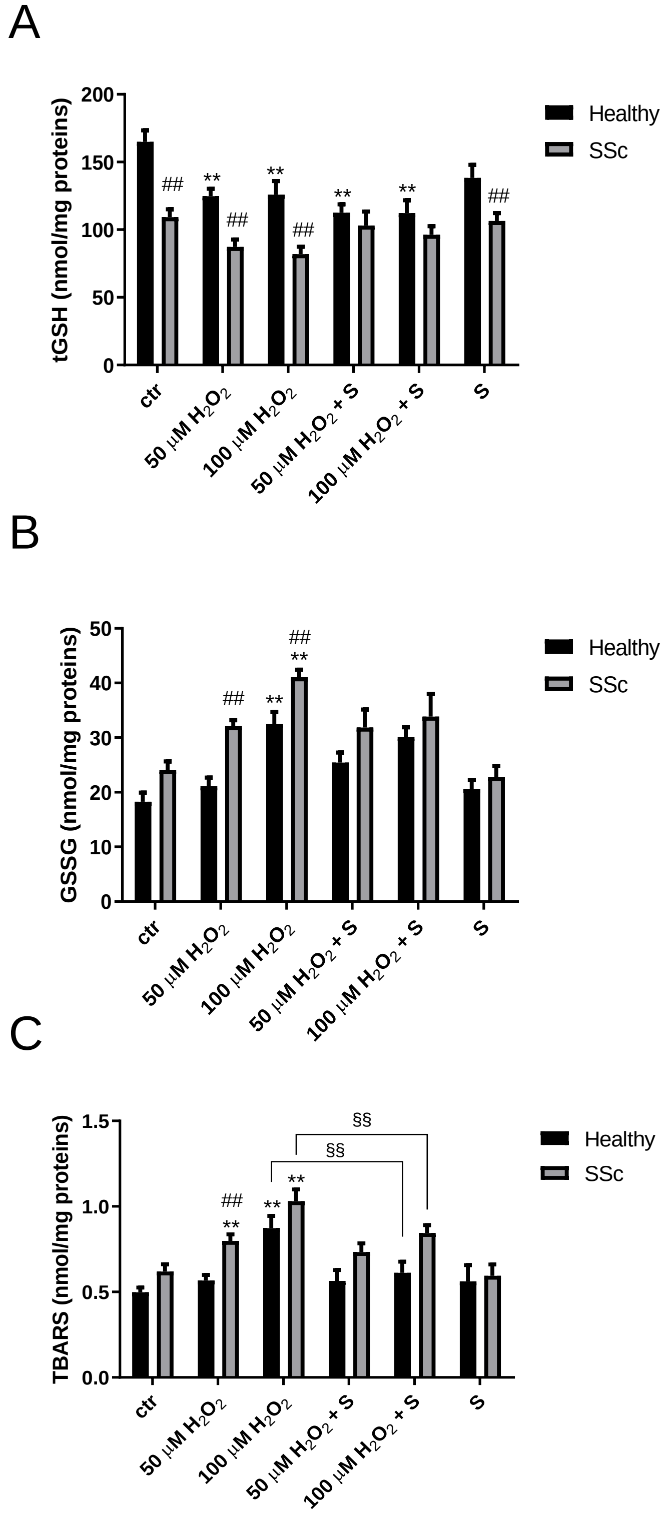

Figure S2. Analysis of redox status in healthy control and SSc fibroblasts treated with hydrogen peroxide. Healthy (black columns) and SSc (grey columns) fibroblasts were treated with 50 and 100μM H_2O_2 in presence or in absence of sildenafil 1μM and analyzed for (A) tGSH content, (B) GSSG, (C) TBARS. Data are expressed as nmol/mg of proteins, mean ± SE. **P<0.01 *vs.* ctr; ##P<0.01 healthy *vs.* SSc; §§P<0.05 H_2O_2 *vs.* H_2O_2 + S. S, sildenafil; tGSH, total glutathione; GSSG, oxidized glutathione; TBARS, thiobarbituric acid reactive substances.
